# Reliability of pedigree-based and genomic evaluations in selected populations

**DOI:** 10.1186/s12711-015-0145-1

**Published:** 2015-08-14

**Authors:** Gregor Gorjanc, Piter Bijma, John M. Hickey

**Affiliations:** The Roslin Institute and Royal (Dick) School of Veterinary Studies, The University of Edinburgh, Easter Bush, Midlothian, Scotland UK; Wageningen University, Animal Breeding and Genomics Centre, Wageningen, The Netherlands

## Abstract

**Background:**

Reliability is an important parameter in breeding. It measures the precision of estimated breeding values (EBV) and, thus, potential response to selection on those EBV. The precision of EBV is commonly measured by relating the prediction error variance (PEV) of EBV to the base population additive genetic variance (base PEV reliability), while the potential for response to selection is commonly measured by the squared correlation between the EBV and breeding values (BV) on selection candidates (reliability of selection). While these two measures are equivalent for unselected populations, they are not equivalent for selected populations. The aim of this study was to quantify the effect of selection on these two measures of reliability and to show how this affects comparison of breeding programs using pedigree-based or genomic evaluations.

**Methods:**

Two scenarios with random and best linear unbiased prediction (BLUP) selection were simulated, where the EBV of selection candidates were estimated using only pedigree, pedigree and phenotype, genome-wide marker genotypes and phenotype, or only genome-wide marker genotypes. The base PEV reliabilities of these EBV were compared to the corresponding reliabilities of selection. Realized genetic selection intensity was evaluated to quantify the potential of selection on the different types of EBV and, thus, to validate differences in reliabilities. Finally, the contribution of different underlying processes to changes in additive genetic variance and reliabilities was quantified.

**Results:**

The simulations showed that, for selected populations, the base PEV reliability substantially overestimates the reliability of selection of EBV that are mainly based on old information from the parental generation, as is the case with pedigree-based prediction. Selection on such EBV gave very low realized genetic selection intensities, confirming the overestimation and importance of genotyping both male and female selection candidates. The two measures of reliability matched when the reductions in additive genetic variance due to the Bulmer effect, selection, and inbreeding were taken into account.

**Conclusions:**

For populations under selection, EBV based on genome-wide information are more valuable than suggested by the comparison of the base PEV reliabilities between the different types of EBV. This implies that genome-wide marker information is undervalued for selected populations and that genotyping un-phenotyped female selection candidates should be reconsidered.

**Electronic supplementary material:**

The online version of this article (doi:10.1186/s12711-015-0145-1) contains supplementary material, which is available to authorized users.

## Background

Selection in livestock breeding programs is commonly based on estimated breeding values (EBV) of selection candidates. In addition to EBV, the variance of prediction errors of EBV (PEV) is also routinely calculated based on the statistical model that is used for genetic evaluation in order to provide a measure of the precision with which the EBV are estimated [1, 2]. PEV for genetic evaluations are routinely produced, either by computationally intensive direct inversion of the left hand side of the mixed model equations or, where this is not possible, by approximations [3–7] or selection index theory [8, 9]. To make interpretation of the precision of published EBV easier for the end user and because of the relationship between reliability and response to selection [10], many breeding programs report the reliability of EBV derived from PEV instead of directly reporting PEV, calculated as 1 minus the ratio between PEV and additive genetic variance. Typically, additive genetic variance in the base population is available and used in calculations, what we will call the base PEV reliability, which quantifies the magnitude of PEV in relation to the base additive genetic variance. This measure of reliability is commonly used to reflect the extent to which EBV may change when more information becomes available, which is particularly relevant in breeding programs with overlapping generations, *e.g.*, in dairy cattle breeding, but much less so in, *e.g*., pig and poultry breeding programs.

Another measure of the reliability of EBV is the squared correlation between breeding values (BV) and EBV of selection candidates. This measure will hereafter be called the reliability of selection because it measures the response to selection that can be obtained when individuals are selected on those EBV, since response to selection is proportional to the accuracy of the EBV, *i.e.*, to the square root of the reliability [10]. The base PEV reliability and the reliability of selection are equivalent for unselected populations (See Appendix) but not for selected populations, because selection reduces additive genetic variance and therefore also the reliability of selection [9, 11–15]. A recent study [16] showed that base PEV reliability may substantially overestimate the reliability of selection for selected populations, and that the equilibrium value of the latter, *i.e.*, the equilibrium reliability, can be predicted from the parameters of unselected populations. The theoretical basis of this overestimation is demonstrated in Additional file 1 [See Additional file 1]. In summary, this overestimation is due to the reduced additive genetic variance among selection candidates in populations under selection and the magnitude of the overestimation varies depending on the information that contributes to the EBV. The overestimation is larger when the EBV depend more on old information from the parental generation than on new information from the current generation. The old information has lower predictive ability for selected populations than for unselected populations, because that information was already used to perform selection of parents and the base PEV reliability does not consider this selection. More specifically, the EBV of selected parents have a reduced variance and a low correlation with the true BV of progeny, which vary between progeny due to recombination and segregation of parental genomes. An example of an extreme case of overestimation of reliability of selection by the base PEV reliability is when the EBV of selection candidates are based on a pedigree prediction, which uses only the old information to estimate the parent average (PA) component of the EBV. A counter example, for which the overestimation is very small is when the EBV are based on a large progeny test, which provides new information to precisely estimate both the PA and the Mendelian sampling (MS) components of the EBV.

Since the base PEV reliability is a measure of the precision of EBV, it is often used as a measure of efficiency when comparing alternative breeding programs, *i.e.*, as a measure of the reliability of selection. If comparisons between the alternative breeding programs that undergo selection are based on the base PEV reliabilities, then the contribution of old information to response to selection will be overestimated and the contribution of new information will be underestimated. With the introduction of genomics, such comparisons have become very common, *e.g.*, comparing the reliability of progeny-tested males and genomically-tested young males [17]. In addition, these comparisons often involve different types of reliabilities: the base PEV reliability for progeny-tested males and the reliability of selection for genomically-tested young males via either forward validation or cross-validation. These two types of reliabilities are not always comparable because the base PEV reliabilities are the expected theoretical values under the assumption of no selection, while validation measures reliability of selection for the analyzed case.

While traditional pedigree-based evaluations are reasonably accurate at estimating the PA component of breeding values, they often provide limited information to estimate the MS component accurately, particularly for young selection candidates. Genomic data provides new information to estimate both the PA and MS components with moderate reliability, which accounts for its usefulness in breeding programs [17–19]. If the benefit of this new (genome-wide marker) information is evaluated using the base PEV reliability, its usefulness in a breeding program may be undervalued, particularly when compared to the value of old information from the parental generation, *i.e.*, the EBV of selected parents [16]. There are potentially many scenarios that need to take the predictive value of old and new information into account when evaluating the usefulness of genome-wide marker information in breeding programs undergoing selection, as for example, the value of collecting genome-wide marker information on un-phenotyped female selection candidates. To date, most breeding programs have predominantly used genome-wide marker information to select un-phenotyped males, but not females. One of the reasons for this is that the perceived improvement in response to selection when selecting un-phenotyped females using genome-wide marker information is limited, *e.g.,* [20–22].

The aim of this research was to quantify the effect of selection on the two measures of reliability for pedigree-based and genomic evaluation of selection candidates, with the following working objectives: (i) to complement the study of Bijma [16] by comparing the base PEV reliability and reliability of selection for pedigree-based and genomic evaluations in populations under selection; (ii) to evaluate the benefit of having genome-wide marker information in such breeding programs; and (iii) to quantify the effect of selection on additive genetic variance and the reliability of selection and compare these obtained values with theoretical equilibrium reliabilities of [16].

## Methods

The effect of selection on the two measures of reliability was quantified using simulated data by comparing a scenario with random selection to a scenario with selection on best linear unbiased prediction (BLUP) EBV. The simulation procedure involved generating genome, pedigree, and phenotype data, which were in turn used in genetic evaluation of selection candidates with the different types of information. The effect of selection on the two measures of reliability was evaluated by: (i) comparing the base PEV reliabilities and reliabilities of selection, (ii) quantifying the realized genetic selection intensities, and (iii) evaluating the reduction of the reliability of selection due to reduction in additive genetic variance and comparing it to the theoretical equilibrium reliabilities. Ten replicates were simulated and all the calculated statistics were summarized with their average and standard deviation or 95 % confidence interval. All calculations were done in R [23] unless otherwise stated.

### Genome

Sequence data were generated for 4000 base haplotypes for each of 30 chromosomes of the genome using the Markovian Coalescent Simulator (MaCS) [24]. The chromosomes were each 100 cM long, comprised 1.0 × 10^8^ base pairs and were simulated using a per site mutation rate of 2.5 × 10^−8^, a per site recombination rate of 1.0 × 10^−8^, and an effective population size (*N*_*e*_) that varied over time, reflecting the estimates for the Holstein cattle population [25]; in the base generation, *N*_*e*_ was equal to 100 and was increased linearly to 1256 at 1000 years ago, 4350 at 10 000 years ago, and 43 500 at 100 000 years ago. A set of 9000 segregating sites were selected at random from the simulated base haplotypes to represent causative loci affecting a complex trait, with a restriction that 300 were sampled from each chromosome. The allele substitution effect at each causative locus (*α*_*i*_) was sampled from a normal distribution with a mean of 0 and standard deviation of 1 divided by the square root of the number of causative loci, *i.e.*, 1/9000. A second sample of 60 000 segregating sites was selected at random as genome-wide markers on a single nucleotide polymorphism (SNP) array, with a restriction that 2000 SNPs were sampled from each chromosome. There was no restriction on the frequency of causal loci or SNPs.

### Pedigree and phenotypes

The base haplotypes were dropped through a simulated pedigree of 25 generations using the AlphaDrop program [26]. Each generation was generated by factorial mating of 20 males and 500 females, with four half-sib progenies per female. Altogether, there were 20 × 25 × 4 = 2000 individuals per generation, of which half were males and half females.

The true BV of an individual was obtained as the sum of all allele substitution effects of the causative loci, accounting for the individuals’ genotype at these loci. The base additive genetic variance was equal to *σ*_*A*,0_^2^ = ***a***^*T*^***a***/(*n* − 1), where ***a*** is a 0 mean vector of BV of the *n* base individuals. Phenotypes were obtained by adding a residual term to the BV. The residual variance was scaled according to the base additive genetic variance to give a heritability that was set to a high value (0.75). Phenotypes were assigned only to males, which resulted in a breeding scheme in which males had a performance record of their own and records on 51 male half sibs, whereas females had records on 52 male half sibs. This setup was used to mimic the level of reliabilities that are commonly achieved in dairy cattle breeding programs with progeny testing, but keeping the size of the simulated population small. The base PEV reliabilities of the different types of EBV from these data matched closely the level of reported reliabilities from real dairy cattle breeding programs, *e.g.*, [17, 20, 22].

### Scenarios

In the random selection scenario (Table 1), each of the 25 generations were simulated by mating 20 males and 500 females that were each selected at random from a set of 1000 selection candidates of each gender. In the BLUP selection scenario, the simulation involved two stages to generate genomes influenced by selection. In the first stage, 10 generations were generated as in the random selection scenario to reach equilibrium in the pedigree information, so that subsequent selection on this information would induce a reduction in additive genetic variance, i.e., the Bulmer effect [11, 15, 16]. In the second stage, each of the 15 generations were simulated by mating 20 males and 500 females that were each selected from a set of 1000 selection candidates of one sex based on BLUP evaluation using pedigree and phenotype information from the current and all previous generations. This procedure provided data to analyze the effect of selection on the two measures of reliability when the Bulmer effect had reached equilibrium, which was conservatively assumed to be reached after five generations of selection. The results confirmed this assumption. Therefore, the data from generations 16 to 25 were in equilibrium and used to analyze the effect of selection on reliabilities, as described in the following.

**Table 1:** Simulation design and data available for analysis

Generation	Purpose	Selection	Genetic evaluation using
1 to 10	Pedigree information	Random	Pedigree
11 to 15	Bulmer equilibrium	Random or BLUP	Pedigree
16 to 20	Training population	Random or BLUP	Pedigree, markers, phenotypes
21 to 25	Validation population	Random or BLUP	Pedigree, markers

### Genetic evaluation

The simulated data were subject to retrospective genetic evaluation of selection candidates in each generation using different combinations of the following information (Table 1): pedigree for 25 generations, 60 000 genome-wide marker genotypes for 5000 males from generations 16 to 20 and for 2500 males and females from generations 21 to 25 (*i.e.*, a random sample of 500 individuals from each generation), and 5000 phenotypes for males from generations 16 to 20. Individuals in generations 21 through 25 had no phenotypes and served as a validation set to show the reduction in reliabilities with each next generation of prediction. To limit the amount of computing, a random sample of 500 validation individuals per generation was taken to represent the whole generation and evaluated using the different types of information.

Genetic evaluation was based on the following standard mixed model [2]:

1 $$ \mathbf{y}=\mathbf{X}\mathbf{b}+\mathbf{Z}\mathbf{a}+\mathbf{e}, $$

where **y** is a vector of phenotype records, **b** is a vector of fixed effects (only intercept was used), **a** ~ *N*(0, **V**_a_) is a vector of BV with an additive genetic covariance matrix **V**_*a*_, **e** ~ *N*(0, **V**_e_) is a vector of residuals with a residual covariance matrix of **V**_e_ = **I***σ*_*E*_^2^, and **X** and **Z** are incidence matrices that link phenotype records to **b** and **a**, respectively. Pedigree and genomic evaluations differed in the specification of the covariance structure for **a**; **V**_a_ = **A***σ*_*A*,0_^2^ for the pedigree model and **V**_a_ = **G***σ*_*A*,0_^2^ for the genomic model, where **A** and **G** are the respective relationship matrices based on pedigree [2] and genome-wide marker genotypes [27]. A complete pedigree with all 25 generations was used when setting up the **A** matrix. All analyses were performed with the assumed known intercept (**b**) and variances (*σ*_*A*,0_^2^ *and σ*_*E*_^2^) to facilitate comparison of reliabilities and to avoid variation in the results due to the estimation of parameters that were not of interest in this study. For this reason, the intercept value was first estimated with model (1) and then reused as a known parameter when estimating **a**.

Using the available data (Table 1), four types of EBV were computed for the selection candidates: (i) EBV_P_ was estimated from pedigree information only, using the pedigree model for all individuals in generations 20 to 25 that were free of phenotypic information from their own performance, collateral relatives, or descendants; (ii) EBV_P__&Y_ was estimated from pedigree and phenotype information, using the pedigree model for males and females in generation 20, in which the males had own performance phenotype records and records on male half-sibs, while the females only had records on male half-sibs; (iii) EBV_M__&Y_ was estimated from genome-wide marker and phenotype information, using the genomic model for males in generation 20 that had an own performance phenotype record; (iv) EBV_M_ was estimated from genome-wide marker information only, using the genomic model for a random sample of validation individuals from generations 21 to 25 that had no phenotype information.

### Reliability

The reliability of selection was calculated as the squared correlation between the EBV and BV for selection candidates. The PEV reliability of an EBV was computed as:

2 $$ \begin{array}{rr}\hfill {R}^2\left({\widehat{a}}_i\right)& \hfill =1-\frac{Var\left({a}_i-{\widehat{a}}_i\right)}{Var\left({a}_i\right)}\end{array}, $$

where *Var*(*a*_*i*_ − *â*_*i*_) is the variance of prediction errors of the EBV of animal *i* (PEV), which was obtained by inverting the coefficient matrix corresponding to the model used (1), and *Var*(*a*_*i*_) is a measure of additive genetic variance *σ*_*A*_^2^ (See Appendix). The base PEV reliability was calculated using equation (2), with *Var*(*a*_*i*_) set to the base additive genetic variance *σ*_*A*,0_^2^ corrected for inbreeding. This correction was applied due to substantial reduction in *σ*_*A*,0_^2^ caused by the deep pedigree and limited number of parents used in the simulation. In addition to this, the PEV reliability was calculated using equation (2) with *Var*(*a*_*i*_) set to different values of additive genetic variance *σ*_*A*_^2^ (See subsection “Variances” for details).

### Realized genetic selection intensity

The realized genetic selection intensity was defined as the selection differential of BV realized by retrospectively selecting the candidates on a particular type of EBV, standardized by *σ*_*A*,0_. This metric was chosen to show the potential for generating response to selection based on the different types of EBV in order to confirm the effect of selection on the reliability of selection. Otherwise, this metric does not provide any additional information beyond the reliability of selection and can be computed only when simulated data is available.

### Variances

To quantify the effect of changes in genetic variance on the reliability of selection, the following variances were computed for each generation: (i) the observed additive genic variance; (ii) the expected additive genic variance; and (iii) the additive genetic variance. Here, the additive genetic variance (*σ*_*A*_^2^) refers to the variance of true breeding values and the additive genic variance (*σ*_*α*_^2^) refers to the additive genetic variance under the assumption of linkage equilibrium between the causative loci, *e.g.*, [10, 28]. The observed additive genic variance in generation *t* (including the base generation) was computed as:

3 $$ {\sigma}_{\alpha, t}^2={\displaystyle \sum }{p}_{i,t}{q}_{i,t}{\alpha}_i^2, $$

where *p*_*i*,*t*_ and *q*_*i*,*t*_ are the allele frequencies in generation *t* and *alpha*_*i*_ is the allele substitution effect of the *i*-th causative locus. Inbreeding changes the additive genic variance and its expectation in generation *t* of a randomly mated finite population was computed as:

4 $$ {\sigma}_{\alpha, t}^2={\displaystyle \sum }{p}_{i,t}{q}_{i,t}{\alpha}_i^2, $$

where *N*_*e*_ is the effective size of the population and ${\overline{F}}_t$ is a mean inbreeding coefficient in generation *t* [29]. The equation (4) was also used to correct for the effect of inbreeding on the additive genetic variance when calculating the base PEV reliability using equation (2). Note that *σ*_*α*,0_^2^ ≈ *σ*_*A*,0_^2^ because the base generation was in linkage equilibrium. The difference between the observed additive genic variance in the base generation (3) and the expected additive genic variance in generation *t* (4) was used to estimate the cumulative change in additive genic variance due to inbreeding up to generation *t*:

5 $$ \varDelta {\sigma}_{\alpha, t,inb}^2={\sigma}_{\alpha, 0}^2-{\sigma}_{\alpha, t,inb}^2, $$

while the difference between the expected and observed additive genic variance in generation *t* was used to estimate the cumulative change in additive genic variance due to selection up to generation *t*:

6 $$ {\sigma}_{\alpha, t,inb}^2={\sigma}_{\alpha, 0}^2{\left(1-\frac{1}{2{N}_e}\right)}^t={\sigma}_{\alpha, 0}^2\left(1-{\overline{F}}_t\right), $$

The total change in the additive genic variance up to generation *t* was therefore equal to:

7 $$ \varDelta {\sigma}_{\alpha, t}^2={\sigma}_{\alpha, 0}^2-{\sigma}_{\alpha, t}^2=\varDelta {\sigma}_{\alpha, t,sel}^2+\varDelta {\sigma}_{\alpha, t,inb}^2. $$

The additive genetic variance in generation *t* (*σ*_*A*,*t*_^2^) was computed as the variance of BV in generation *t* prior to any selection within that generation. The difference between the additive genic and the additive genetic variances in the BLUP selection scenario was used to estimate the gametic phase disequilibrium covariance due to the Bulmer effect [11]:

8 $$ {\overline{F}}_t $$

These variances (3) to (8) were used to gradually correct (reduce) the base additive genetic variance and calculate the PEV reliability based on these corrected values to analyze the effect of the different underlying processes on the reduction of the reliability of selection in comparison to the base PEV reliability. In addition, the theoretical expectation of reliability in selected populations, referred to as equilibrium reliabilities, were also calculated for comparison to the base PEV reliabilities corrected for inbreeding (see above) and the proportion of the selected individuals, *i.e.*, 2 % selected males and 50 % selected females [16].

### Analysis

The focal generations for comparison of the base PEV reliabilities and reliabilities of selection and realized genetic selection intensities were generation 20 based on phenotyped males and un-phenotyped females and generations 21 to 25 based on un-phenotyped individuals of both sexes. Changes in the variances were evaluated across all generations. The effect of changes in variances on the PEV reliability and the reliability of selection was analyzed in detail in generations 20 and 21 and compared to the equilibrium reliabilities.

## Results

### Reliability

In the random selection scenario, the base PEV reliabilities and reliabilities of selection were equal, within the bounds of sampling, for both the pedigree model and the genomic model (Table 2) and, therefore, only base PEV reliabilities will be described. In general, reliabilities increased with more information on the MS component of BV. The base PEV reliability of EBV_P_ was equal to 27 % in generations 20 and 21 and decreased each generation to 0 % in generation 25. The base PEV reliability of EBV_P__&Y_ in generation 20 was higher than that of EBV_P_ due to the availability of phenotypic information (35 % for females and 76 % for males). The base PEV reliability of EBV_M__&Y_ was even higher due to the availability of genome-wide marker and phenotype information (84 % in generation 20). The base PEV reliability of EBV_M_ decreased at a slower rate over generations than that of EBV_P,_, *i.e.*, it was equal to 67 % in generation 21 and decreased to 53 % in generation 25.

**Table 2:** Prediction error variance (PEV) reliability and reliability of selection (%)^a^ of different types of estimates of breeding values (EBV)^b^ by scenario and generation

	PEV reliability			Reliability of selection		
	EBV_P_	EBV_P__&Y_	EBV_M_	EBV_P_	EBV_P__&Y_	EBV_M_
Generation^c^: random selection						
20_f_	27_1_	35_1_	/	25_7_	32_6_	/
20_m_	27_1_	76_1_	84_1_	25_7_	75_2_	84_2_
21	27_1_	/	67_1_	29_6_	/	70_3_
22	13_1_	/	61_1_	11_3_	/	63_4_
23	5_1_	/	58_1_	6_5_	/	58_7_
24	1_1_	/	55_1_	4_2_	/	54_7_
25	0_1_	/	53_1_	2_1_	/	56_5_
Generation^c^: BLUP selection						
20_f_	27_1_	35_1_	/	3_3_	13_3_	
20_m_	27_1_	75_1_	85_1_	3_3_	67_1_	80_1_
21	27_1_	/	70_1_	3_2_	/	63_3_
22	12_1_	/	65_1_	0_1_	/	59_3_
23	3_1_	/	62_1_	0_1_	/	54_3_
24	0_1_	/	59_1_	0_1_	/	51_4_
25	0_1_	/	57_1_	1_1_	/	50_4_

^a^Average and standard error (as shown as subscripts)

^b^EBV_P_ = estimates of breeding value based on pedigree information; EBV_P__&Y_ = estimates of breeding value based on pedigree and phenotype information; EBV_M_ = estimates of breeding value based on marker information (in generation 20 estimates of breeding value are based on marker and phenotype information, *i.e.*, EBV_M__&Y_)

^c^in generation 20, the results are presented separately for females (f) and males (m), because males have own phenotype records, whereas females do not

In the BLUP selection scenario, the base PEV reliabilities followed the same pattern as in the random selection scenario. However, the reliabilities of selection were consistently lower than the base PEV reliabilities, especially for EBV with a large dependency on PA information (Table 2), which shows that the base PEV reliabilities overestimated the reliabilities of selection in this scenario. The ratio of the reliability of selection to the base PEV reliability in generation 20 was equal to 0.11 for EBV_P_, 0.37 for EBV_P__&Y_ for females, 0.89 for EBV_P__&Y_ for males, and 0.94 for EBV_M__&Y_. In generation 21, the ratio of reliabilities for EBV_P_ was equal to 0.11 and 0.00 in the following generations, while for EBV_M_ the ratio was equal to 0.90, 0.91, 0.87, 0.86, and 0.88 in generations 21 to 25, respectively (Table 2).

Comparison of reliabilities of the different types of EBV obtained with the BLUP selection scenario showed that genomic prediction had a greater advantage over pedigree prediction when based on the reliability of selection than when based on base PEV reliability. For example, the difference between the reliability of genomic and pedigree predictions in generation 21 was 17 % larger when based on reliability of selection than when based on base PEV reliability, which indicates that genotyping un-phenotyped females might be more valuable than previously suggested, *e.g.,* [20–22].

### Realized genetic selection intensity

To confirm differences between base PEV reliabilities and reliabilities of selection, selection on the different types of EBV was compared in terms of realized genetic selection intensities of BV that could have been achieved if candidates were selected on those EBV. In general, realized genetic selection intensities reflected the reliabilities of selection for both the random selection scenario and the BLUP selection scenario and confirmed that base PEV reliabilities overestimate reliabilities of selection in the BLUP selection scenario. Differences between the realized genetic selection intensities were smaller than between the two measures of reliability, because realized genetic selection intensities are proportional to the accuracy of selection, *i.e.*, to the square root of reliability of selection.

In the random selection scenario, selecting candidates directly on true BV gave realized genetic selection intensities that ranged from 0.73 to 0.76 with 50 % selected and from 2.16 to 2.24 with 2 % selected (Table 3). Selection on EBV_P_ gave the lowest realized genetic selection intensities, which ranged from 0.19 to 0.22 with 50 % selected and from 0.55 to 0.60 with 2 % selected in generations 20 and 21. These values practically decreased by 50 % in each next generation due to the low predictive ability of EBV_P_. Selection on EBV_P__&Y_ gave higher realized genetic selection intensities than selection on EBV_P_ due to the higher reliabilities of EBV when based on phenotype information on full-sibs and half-sibs for females, as well as own performance records for males. Realized genetic selection intensities with EBV_P__&Y_ were equal to 0.24 and 0.55 with 50 % selected, and to 0.75 and 1.67 with 2 % selected, respectively. Selection on EBV_M__&Y_ gave the highest realized genetic selection intensity due to the use of genome-wide marker and phenotype information. In generation 20, the realized genetic selection intensities for EBV_P__&Y_ and EBV_M__&Y_ were equal to 0.55 and 0.62 with 50 % selected and to 1.67 and 1.90 with 2 % selected, respectively. In the later generations, selecting on EBV_M_ gave more than half of the realized genetic selection intensity compared to selecting directly on true BV.

**Table 3:** Realized genetic selection intensity^a^ when selecting on true breeding value (BV) or different types of estimates of breeding values (EBV)^b^ by proportion selected, scenario, and generation

	Proportion selected = 50 %				Proportion selected = 2 %			
	BV	EBV_P_	EBV_P__&Y_	EBV_M_	BV	EBV_P_	EBV_P__&Y_	EBV_M_
Generation^c^: random selection								
20_f_	0.73_0.04_	0.19_0.05_	0.24_0.06_	/	2.20_0.19_	0.60_0.21_	0.75_0.19_	/
20_m_	0.74_0.04_	0.19_0.05_	0.55_0.04_	0.62_0.04_	2.23_0.17_	0.55_0.25_	1.67_0.16_	1.90_0.15_
21	0.75_0.04_	0.22_0.04_	/	0.54_0.04_	2.23_0.21_	0.60_0.22_	/	1.65_0.19_
22	0.76_0.03_	0.09_0.02_	/	0.48_0.03_	2.24_0.18_	0.27_0.09_	/	1.45_0.13_
23	0.73_0.04_	0.05_0.03_	/	0.44_0.05_	2.21_0.28_	0.13_0.11_	/	1.35_0.26_
24	0.73_0.06_	0.02_0.01_	/	0.41_0.05_	2.16_0.15_	0.07_0.06_	/	1.22_0.16_
25	0.74_0.04_	0.01_0.01_	/	0.42_0.03_	2.17_0.15_	0.03_0.06_	/	1.27_0.14_
Generation^c^: BLUP selection								
20_f_	0.61_0.02_	0.02_0.01_	0.08_0.01_	/	1.84_0.10_	0.09_0.03_	0.26_0.08_	/
20_m_	0.62_0.02_	0.02_0.01_	0.41_0.02_	0.51_0.02_	1.87_0.11_	0.10_0.07_	1.26_0.06_	1.56_0.09_
21	0.60_0.03_	0.02_0.01_	/	0.38_0.03_	1.87_0.17_	0.08_0.06_	/	1.22_0.14_
22	0.61_0.02_	0.00_0.01_	/	0.38_0.02_	1.83_0.10_	0.01_0.04_	/	1.18_0.14_
23	0.59_0.03_	0.00_0.01_	/	0.37_0.03_	1.80_0.14_	−0.01_0.02_	/	1.11_0.11_
24	0.58_0.02_	0.00_0.01_	/	0.36_0.03_	1.73_0.12_	0.00_0.01_	/	1.06_0.17_
25	0.58_0.02_	0.00_0.01_	/	0.34_0.02_	1.74_0.12_	0.00_0.02_	/	1.03_0.17_

^a^Average and standard error (as subscript)

^b^EBV_P_ = estimates of breeding value based on pedigree information; EBV_P__&Y_ = estimates of breeding value based on pedigree and phenotype information, EBV_M_ = estimates of breeding value based on marker information (in generation 20 estimates of breeding value are based on marker and phenotype information, *i.e.*, EBV_M__&Y_)

^c^in generation 20 the results are presented separately for females (f) and males (m), because males have own performance records, whereas females do not

In the BLUP selection scenario, selection on true BV gave realized genetic selection intensities that ranged from 0.58 to 0.62 with 50 % selected and from 1.74 to 1.87 with 2 % selected and remained constant (within the bounds of sampling) over all generations (Table 3). These results in the BLUP selection scenario are between 16 and 22 % lower than for the random selection scenario, with an increasing trend over time. Selection on EBV_P_ gave a realized genetic selection intensity of only 0.02 with 50 % selected and between 0.09 and 0.10 with 2 % selected in generation 20, and dropped to 0 in the later generations much more quickly than with the random selection scenario. These realized intensities with EBV_P_ were more than 80 % lower than with the random selection scenario. With EBV_P__&Y_, the reduction of realized genetic selection intensity in comparison to the random selection scenario was 66 % for females and 25 % for males. With EBV_M__&Y_ and EBV_M_, the reduction of realized genetic selection intensity was between 12 and 30 %, with the largest difference observed in generation 21, which was the first generation of prediction without phenotype information.

### Changes in variances and effect on reliability

**Fig. 1:**
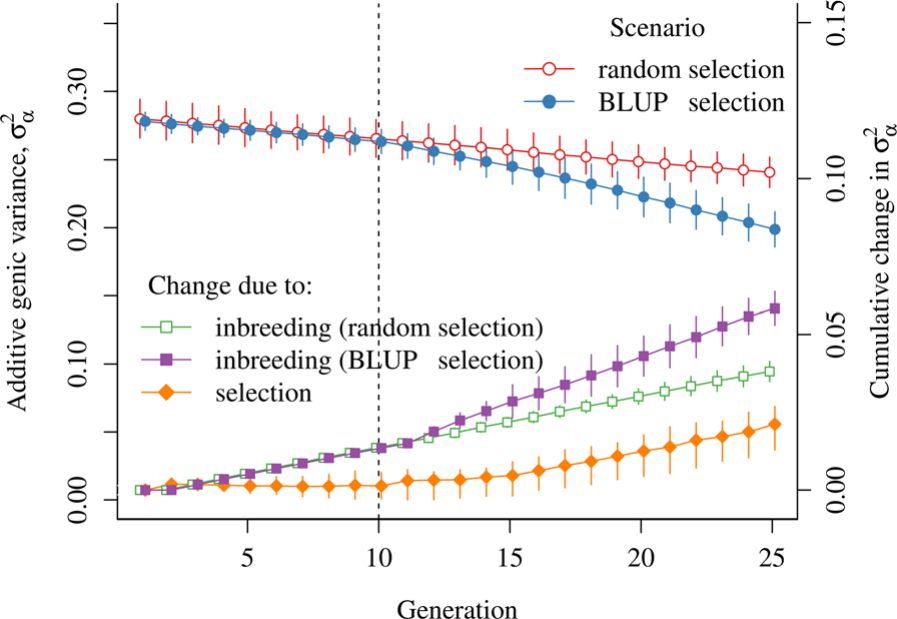
Additive genic variance (*σ*_*α*_^2^) and changes due to inbreeding and selection by scenario and generation. Average values with 95 % confidence intervals are presented

Additive genic variance decreased with each generation in both the random and BLUP selection scenarios, although the reduction was larger with the BLUP selection scenario (Fig. 1). Additive genic variance in the base generation was equal to 0.28 with both scenarios and by generation 20 it was reduced to 0.25 with the random selection scenario and to 0.22 with the BLUP selection scenario. These reductions were mainly caused by inbreeding and were quantified by subtracting the expected additive genic variance under the finite population model from the base generation value (5). The reduction caused by inbreeding up to generation 20 was equal to 0.03 with the random selection scenario and 0.045 with the BLUP selection scenario. The remaining loss of 0.015 in genic variance with the BLUP selection scenario was attributed to the effect of selection.

**Fig. 2:**
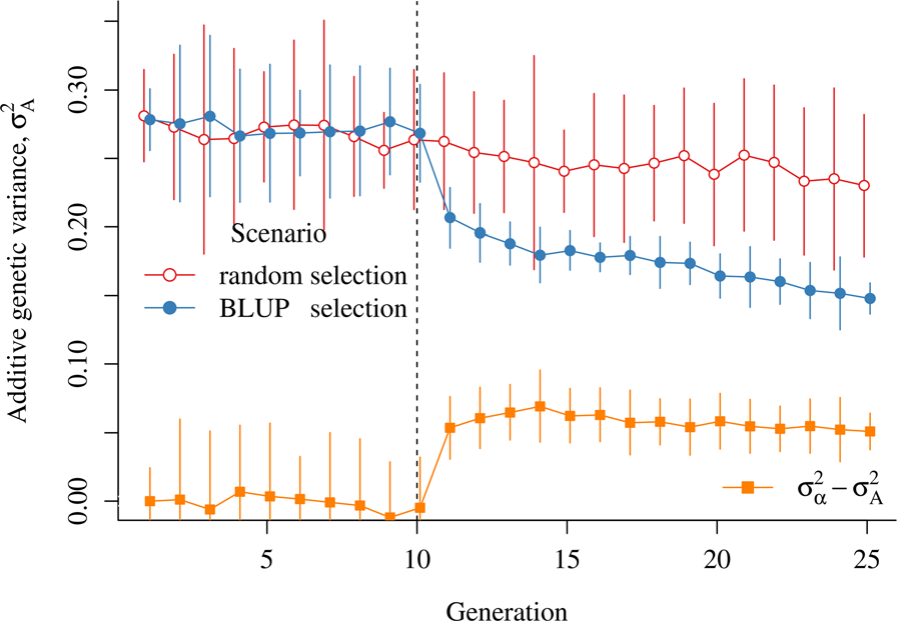
Additive genetic variance (*σ*_*A*_^2^) and Bulmer effect (*σ*_*α*_^2^ − *σ*_*A*_^2^) by scenario and generation. Average values with 95 % confidence intervals are presented

For both scenarios, the additive genetic variance also decreased with each generation, but with a significant change in generation 10 when selection on EBV was introduced in the BLUP selection scenario (Fig. 2). Additive genetic variance was equal to 0.28 in the base generation with both scenarios and by generation 10, it decreased to 0.26 for both scenarios because of inbreeding. Introduction of selection in generation 10 reduced the additive genetic variance to 0.21 in generation 11, while the additive genic variance was equal to 0.26. The difference between these two variances gave an estimate of −0.05 for the gametic phase disequilibrium covariance. By generation 20, the additive genetic variance was further reduced to 0.16. The overall reduction of the base additive genetic variance (0.28) was due half to the Bulmer effect (0.06) and half to loss in additive genic variance caused by inbreeding (0.045) and selection (0.015). In the random selection scenario, the additive genetic variance in generation 20 was equal to 0.24, which was equal to additive genic variance within the bounds of sampling.

**Fig. 3:**
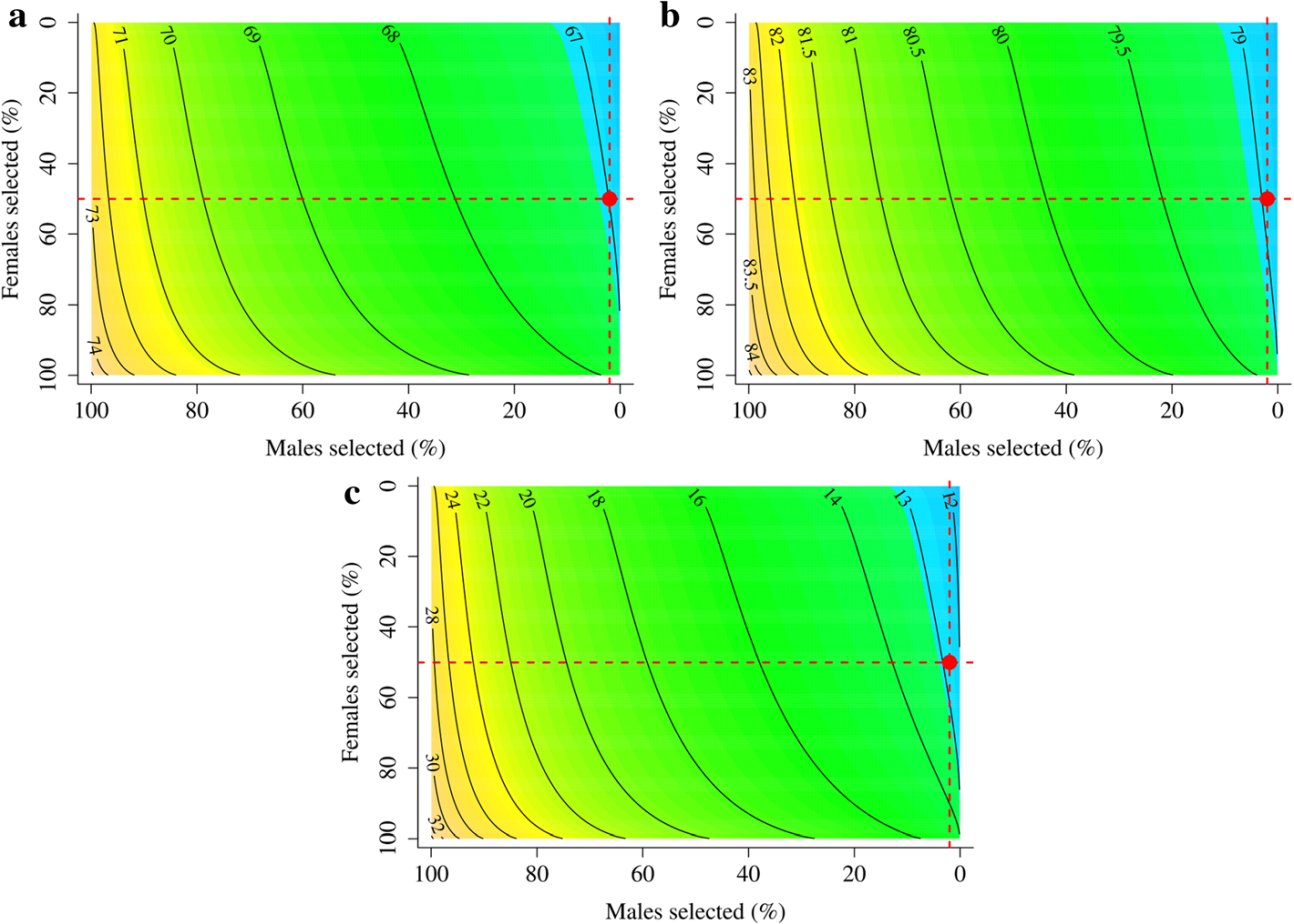
Equilibrium reliability and reliability of selection of different types of estimated breeding values in generation 20. Breeding values estimated using (**a**) pedigree and phenotype information in males (EBV_P__&Y,m_), (**b**) marker and phenotype information in males (EBV_M__&Y,m_), and (**c**) pedigree and phenotype information in females (EBV_P__&Y,f_). Equilibrium reliabilities are shown with contours, as a function of the proportions of males and females selected, while reliability of selection is shown as a point at the proportions selected used in this study

**Fig. 4:**
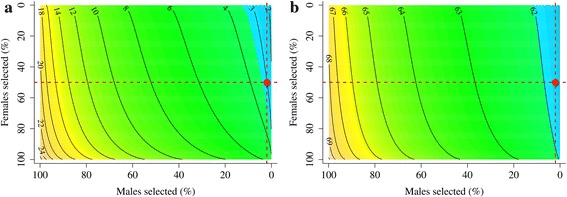
Equilibrium reliability and reliability of selection of different types of estimated breeding values in generation 21. Breeding values estimated (predicted) using (**a**) pedigree information (EBV_P_) and (**b**) marker information (EBV_M_). Equilibrium reliabilities are shown with contours as a function of the proportions of males and females selected, while reliability of selection is shown as a point at the proportions selected used in this study

The effect of changes in variances (Figs. 1 and 2) on the reliability of selection was quantified in detail in generations 20 and 21 by calculating the PEV reliability with different values of additive genetic variance (Table 4). In the random selection scenario, the reliability of selection tended to be lower than the base PEV reliabilities. Taking into account the reduction in variance due to inbreeding, or using the additive genetic variance from generation 20 or 21, gave a PEV reliability that matched the reliability of selection within the bounds of sampling. In the BLUP selection scenario, the base PEV reliabilities considerably overestimated the reliability of selection, as previously noted (Table 2). This overestimation was, due to the reduction in additive genetic variance, as caused by several underlying processes (Table 4). Inbreeding, and to a small extent selection, reduced the additive genic variance and therefore also the additive genetic variance by changing the allele frequencies of causative loci. More importantly, the additive genetic variance was also reduced by the generation of gametic phase disequilibrium between the causative loci by selection, *i.e.*, the Bulmer effect. These reductions in additive genetic variance due to inbreeding, selection, and the Bulmer effect were used to gradually reduce the base additive genetic variance to the additive genetic variance in generation 20 or 21 and to recalculate the PEV reliabilities for each reduction. The resulting PEV reliabilities matched the reliability of selection within the bounds of sampling. These results not only show which processes contribute to the reduction of the reliability of selection in selected populations but also that the base PEV reliabilities overestimate the reliability of selection in such populations by using the base additive genetic variance instead of actual additive genetic variance of selection candidates. Finally, the equilibrium reliabilities matched the reliability of selection for EBV_P_ and EBV_P__&Y_ (Table 4 and Figs. 3 and 4, while there were minor discrepancies for EBV_M__&Y_ and EBV_M_. Figures 3 and 4 show contours of equilibrium reliabilities for the different proportion of selected males and females and a dot for the reliability of selection obtained in this study (Table 4). The discrepancies for EBV_M__&Y_ and EBV_M_ arose because, in this study, selection was on EBV_P__&Y_ and calculating the equilibrium reliabilities with the higher EBV_M__&Y_ or EBV_M_ base PEV reliabilities, as if selection was on the EBV_M__&Y_ or EBV_M,_ leads to underestimation of the equilibrium reliabilities. Changing the proportion of selected males and females when calculating the equilibrium reliability for EBV_P__&Y_ and EBV_M__&Y_ in generation 20 (Fig. 3) and for EBV_P_ and EBV_M_ in generation 21 (Fig. 4) showed that the observed base PEV reliabilities were recovered when selection was absent, *i.e.*, the equilibrium reliabilities from the bottom-left corners of Figs. 3 and 4 matched the base PEV reliabilities corrected for inbreeding in Table 4.

**Table 4:** Prediction error variance (PEV) reliabilities^a^ based on different measures of additive genetic variance^b^ (**V**_***A***_), reliability of selection^a^, and equilibrium reliabilities^a^ (%) of different types of estimates of breeding values (EBV)^c^ by scenario in generations 20 and 21

Reliability	EBV_P,20_	EBV_P,21_	EBV_P__&Y,20,f_	EBV_P__&Y,20,m_	EBV_M__&Y,20_	EBV_M,21_
Random selection						
PEV						
*σ*_*A*_^2^ = *σ*_*A*,0_^2^	35_1_	36_1_	42_1_	79_1_	86_1_	71_1_
*σ*_*A*_^2^ = *σ*_*A*,0_^2^ − Δ*σ*_*α*,*t*,*inb*_^2^	27_1_	27_1_	35_1_	76_1_	84_1_	67_1_
*σ*_*A*_^2^ = *σ*_*A*,*t*_^2^	23_1_	28_1_	31_6_	75_1_	83_2_	67_4_
Reliability of selection	25_7_	29_6_	32_6_	75_2_	84_2_	70_3_
BLUP selection						
PEV						
*σ*_*A*_^2^ = *σ*_*A*,0_^2^	39_1_	39_1_	45_1_	79_1_	87_1_	75_1_
*σ*_*A*_^2^ = *σ*_*A*,0_^2^ − Δ*σ*_*α*,*t*,*sel*_^2^	36_1_	36_1_	43_1_	78_1_	87_1_	74_1_
*σ*_*A*_^2^ = *σ*_*A*,0_^2^ − Δ*σ*_*α*,*t*,*inb*_^2^	27_1_	27_1_	35_1_	75_1_	85_1_	70_1_
*σ*_*A*_^2^ = *σ*_*A*,0_^2^ − Δ*σ*_*GD*,*t*_^2^	22_4_	24_4_	30_4_	74_1_	84_1_	69_2_
*σ*_*A*_^2^ = *σ*_*A*,0_^2^ − Δ*σ*_*α*,*t*_^2^ − Δ*σ*_*GD*,*t*_^2^	−5_9_	−4_8_	7_8_	65_3_	78_2_	58_3_
*σ*_*A*_^2^ = *σ*_*A*,*t*_^2^	−4_5_	−4_7_	7_4_	65_2_	78_1_	58_3_
Reliability of selection	3_3_	3_2_	13_3_	67_1_	80_1_	63_3_
Equilibrium	3_1_	3_1_	13_1_	67_1_	79_1_	62_1_

^a^Average and standard error (as subscript)

^b^ *σ*_*A*,0_^2^ **=** base additive genetic variance; Δ*σ*_*α*,*t*,*inb*_^2^ **=** change in additive genic variance due to inbreeding from base to generation *t* (5); Δ*σ*_*α*,*t*,*sel*_^2^ **=** change in additive genic variance due to selection from base to generation *t* (6); Δ*σ*_*α*,*t*_^2^ = Δ*σ*_*α*,*t*,*inb*_^2^ + Δ*σ*_*α*,*t*,*sel*_^2^ **=** change in additive genic variance due to inbreeding and selection from base to generation *t* (7); Δ*σ*_*GD*,*t*_^2^ **=** change in additive genetic variance due to the Bulmer effect (8); *σ*_*A*,*t*_^2^ = additive genetic variance in generation *t*

^c^EBV_P_ = estimates of breeding value based on pedigree information; EBV_P__&Y_ = estimates of breeding value based on pedigree and phenotype information; EBV_M_ = estimates of breeding value based on marker information; EBV_M__&Y_ = estimates of breeding value based on marker and phenotype information; other subscripts denote generation (20 and 21) and a group of males (m) or females (f)

## Discussion

Reliability is important in breeding because it measures the potential for response to selection in a breeding program. The results of this study show that, in populations under selection, reliability computed from PEV and the base additive genetic variance (base PEV reliability) is not equal to the squared correlation between the EBV and BV in selection candidates (reliability of selection), with which potential for response to selection is measured. The difference between these two measures of reliability arises from their different scopes of interpretation. The base PEV reliability overestimates the reliability of selection in selected populations because it is computed from PEV and the base additive genetic variance. The latter describes genetic variation in the base population and not in the selection candidates. As shown in this study, this overestimation can be mitigated either by calculating the PEV reliability based on the reduced additive genetic variance of the selection candidates or by using theoretical equilibrium reliabilities. It was also shown that the degree of overestimation differs between types of EBV and that this has important consequences when breeding schemes and genotyping strategies are compared based on the base PEV reliability; in particular when the base PEV reliability of pedigree prediction is compared to that of other types of EBV.

### Reliability in selected populations

The following example illustrates that selection reduces the reliability of selection and that this effect differs between types of EBV. Selecting the parents of the next generation on any type of EBV reduces the variance of these EBV, which are in turn used to obtain pedigree predictions (EBV_P_) of the progeny. In the extreme case, selecting and mating only two parents (with EBV_1_ and EBV_2_ and corresponding base PEV reliabilities ${R}_{EB{V}_1}^2$ and ${R}_{EB{V}_2}^2\Big)$ would create a new generation for which all individuals have the same pedigree prediction, $EB{V}_P=\frac{1}{2}\left( EB{V}_1+ EB{V}_2\right),$ although there would be variation in their BV due to the Mendelian sampling of parental genomes. In such a situation, the EBV_P_ has no predictive ability to differentiate between individuals, although the base PEV reliability of *EBV*_*P*_ would be greater than 0, *i.e.*, ${R}_{EB{V}_P}^2\ge \frac{1}{4}\left({R}_{EB{V}_1}^2+{R}_{EB{V}_2}^2\right)$ [See Additional file 1]. Consequently, these EBV_P_ have no potential to generate response to selection if selection is carried out among progeny. In contrast, genomic predictions (EBV_M_) for these individuals would have some predictive ability and potential to generate response to selection, because genome-wide markers provide new information to estimate both the PA and MS component of EBV for each individual [17–19], which can then be differentiated. However, in selected populations, the predictive ability of EBV_M_ is also overestimated by the base PEV reliability, albeit less so than for EBV_P_.

A detailed illustration on how selection reduces the reliability of selection and how this effect differs between types of EBV is in Additional file 1 [See Additional file 1]. In summary, selection of parents reduces the variance of BV (*i.e.*, additive genetic variance) in progeny but in particular the variance of EBV_P_ in progeny. The reduction of additive genetic variance in progeny reduces the reliability of selection because the unchanged precision of EBV coupled with a smaller variation in BV make it more difficult to differentiate between individuals. The reduction in variance of EBV_P_ in progeny reduces the reliability of selection because EBV_P_ only predicts the PA component of BV and with increasing selection in parents, the predictive ability of EBV_P_ decreases, as illustrated previously. The reduced additive genetic variance in progeny has the same effect on the reliability of selection for any type of EBV. In contrast, the reduced variance of EBV_P_ in progeny has a different effect on the reliability of selection for different types of EBV and is larger for EBV that are primarily based on the PA component and smaller for EBV that are primarily based on the MS component.

These illustrations indicate that the base PEV reliability overestimates the reliability of selection because it does not take into account the effect of selection on variances. The expression for the base PEV reliability involves PEV and the base additive genetic variance. Selection does not affect the PEV [1, 13] but it does affect the additive genetic variance. It causes a reduction in the additive genetic variance that should be taken into account if the PEV reliability is to be used as a measure of the reliability of selection. The rationale behind the expression for the base PEV reliability derives from the PEV being the (posterior) variance of BV conditional on the observed phenotypic information and the base additive genetic variance being the (prior) unconditional variance of BV in the base population. Relating this posterior to the prior quantifies the amount by which the uncertainty in BV is reduced after phenotypic information has been collected [2]. While the base additive genetic variance must be used when calculating EBV and PEV [2], which unconditional variance of BV should be used when calculating the PEV reliability depends on the scope of interpretation. If the aim is to measure the reliability of selection among parents and progeny, then the PEV reliability should be calculated based on the additive genetic variance in parents. However, if the aim is to measure the reliability of selection among progeny, as in the present study, then the PEV reliability should be calculated based on the additive genetic variance in progeny. When the scope of interpretation is not taken into account, the PEV reliability can overestimate the reliability of selection. The amount of overestimation depends on the type of EBV, its base PEV reliability, and the intensity of selection, which determines how much additive genetic variance has been lost over the generations of selection [16].

Therefore, if the PEV reliability is used as a measure of the reliability of selection, it should be computed based on the additive genetic variance of selection candidates. However, this is often not possible because the additive genetic variance for sets of individuals is usually unknown in real populations and its estimation is computationally demanding [13]. In addition, there is usually no clear definition of the generation or groups of individuals of interest in livestock populations, which complicates estimation even more. In such situations, the base additive genetic variance may be the only estimate available and therefore the base PEV reliabilities can only be used as a measure of precision of EBV in relation to the base population variation and not as a measure of the reliability of selection to compare breeding schemes. However, the difference between these two measures of reliability can be predicted using the equilibrium reliabilities calculated from the base PEV reliabilities and the proportions selected among males and females [16]. As shown in this study, the equilibrium reliabilities matched the reliability of selection for any type of EBV, which confirms the utility of theoretical expressions to calculate the equilibrium reliability [16].

In this study, the reduction of additive genetic variance across generations was caused by three processes: the initial cycles of selection caused changes in gametic phase disequilibrium (*i.e.*, the Bulmer effect), and inbreeding and selection caused changes in allele frequencies. The Bulmer effect was responsible for 50 % of the loss of variance, while changes in allele frequencies due to inbreeding and selection were responsible for 37.5, and 12.5 % of the loss of variance, respectively. The theoretical expressions for the equilibrium reliability derived in [16] only account for the reduction in variance due to the Bulmer effect, and not for reductions due to changes in allele frequencies resulting from inbreeding and selection. However, our study demonstrates that the Bulmer effect is the largest source of reduction in variance. In addition, the expected loss of additive genetic variance in finite populations [29] can be used to account for the effect of inbreeding on variance. The effect of inbreeding was substantial in this study, because of the deep pedigree and a small number of parents. In more typical scenarios, the pedigree is not as deep, which suggests that the impact of reduction in additive genetic variance due to selection changing allele frequency would also be smaller than in this study.

### Implications for comparison of breeding programs

The difference between the base PEV reliabilities and the reliability of selection has important consequences for the design of breeding programs using genome-wide marker information. Genome-wide marker information is often considered to be of much lower value for un-phenotyped females than for males. This perception is in part due to the smaller impact that females have on the next generation, but also due to the relatively small difference between the base PEV reliability of EBV_P_ or EBV_P__&Y_ and the base PEV or validation reliabilities of EBV_M_. For example, in the BLUP selection scenario used in this study, the base PEV reliabilities of EBV_P_ and EBV_M_ in generation 21 were equal to 27 and 70 %, respectively, with an absolute difference of 43 %. Several studies have derived the value of genotyping un-phenotyped females on the basis of gains in reliability, while accounting for cost of genotyping and raising replacement females, *e.g.*, [20–22]. However, our results show that the gain in reliability of selection is much higher than expected from comparison of the base PEV reliabilities; in generation 21, reliability of selection was 3 % for EBV_P_ and 63 % for EBV_M_, with an absolute difference of 60 %. This large difference demonstrates that there is more value in genotyping un-phenotyped females in selected populations than previously reported. This was further demonstrated by measuring the realized genetic selection intensity for the different types of EBV; in generation 21 of the BLUP selection scenario, selecting 50 % of selection candidates gave realized genetic selection intensity of 0.02 when selecting on EBV_P_ and of 0.38 when selecting on EBV_M_. These results clearly show the benefit of investing in genotyping un-phenotyped females. With increased selection intensity, the effect of selection on realized genetic selection intensity was even more pronounced due to further reductions of the base PEV reliability of EBV_P_. Comparing the predictive abilities of EBV_P_ and EBV_M_ is, in some sense, a comparison of extremes. Smaller but still significant differences can be expected when the EBV of selection candidates have a large dependency on information from the parental generation. Failing to take the effect of selection on additive genetic variance into account, can overstate the reliability of selection on such EBV in comparison with EBV_M_ [15, 16]. This is not an issue when the comparison of predictive abilities of EBV_P_ or EBV_P__&Y_ and EBV_M_ are all based on validation correlations among selection candidates.

## Conclusions

Selection reduces genetic variance and the reliability of selection, which is usually not accounted for when the base additive genetic variance is used to calculate base PEV reliabilities. This reduction in reliability of selection is more pronounced for EBV that are based mainly on information from the parental generation. An extreme example of this is when EBV are based solely on parent average. This implies that the genome-wide marker information has been undervalued in populations that are under selection, and that genotyping un-phenotyped females must be reconsidered.

## Appendix

### Equivalence between base PEV reliability and reliability of selection in unselected populations

The purpose of this appendix is to show that the two measures of reliability, the base PEV reliability and the reliability of selection, are equivalent for unselected populations. This is first shown by defining the reliability of selection as the squared correlation between the true breeding values (BV) and estimated breeding values (EBV) in an unselected population [10], and then by demonstrating that this is equivalent to the commonly used expression to compute the base PEV reliability [1, 2, 13].

The reliability of selection is defined as a squared correlation between the BV (**a**) and EBV (**â**) for selection candidates. This value is commonly presented as a single value for a group of individuals [10], which is likely based on the intuition of obtaining the sample correlation between a vector of BV and a vector of EBV. However, expression for the correlation between two vectors results in a matrix of correlations **C**_*â*,*a*_ and squaring its elements via the Hadamard product (∘) gives a matrix of squared correlations:

A1 $$ \begin{array}{rr}\hfill Corr\left(\mathbf{a},{\widehat{\mathbf{a}}}^{\mathrm{T}}\right)={\mathbf{C}}_{\mathrm{a},\widehat{\mathrm{a}}}& \hfill = diag{\left(Var\left(\mathbf{a}\right)\right)}^{-\frac{1}{2}}Cov\left(\mathbf{a},{\widehat{\mathbf{a}}}^{\mathrm{T}}\right) diag{\left(Var\left({\widehat{\mathbf{a}}}^T\right)\right)}^{-\frac{1}{2}},\\ {}\hfill & = diag{\left({\mathbf{V}}_{\mathrm{a}}\right)}^{-\frac{1}{2}}{\mathbf{V}}_{\mathrm{a},\widehat{\mathrm{a}}} diag{\left({\mathbf{V}}_{\widehat{a}}\right)}^{-\frac{1}{2}},\hfill \\ {}\hfill {R}^2\left(\widehat{\mathbf{a}}\right)& \begin{array}{ll}=\hfill & \hfill {\mathbf{C}}_{\mathrm{a},\widehat{\mathrm{a}}}\circ {\mathbf{C}}_{\mathrm{a},\widehat{\mathrm{a}}},\end{array}\hfill \end{array} $$

where the diagonal elements are the squared correlation between BV and EBV for each individual, *i.e.*, the reliability for each individual, while the off-diagonal elements are the squared correlation between BV of one individual and EBV of another individual, *i.e.*, the “co-reliability” for each pair of individuals. In the expression (A1), the notation *diag*(**X**) indicates a diagonal matrix with the diagonal equal to the diagonal of matrix **X**. If the evaluated individuals were unrelated and all of them had a single own phenotype pre-corrected for any other effect without error, then the reliability of this evaluation would be the same for all individuals, *R*^2^, and (A1) could be written as *R*^2^(**â**) = **I***R*^2^, which is in line with the common usage [10]. However, in real applications, evaluated individuals are related, they might have different amounts of information, and phenotypes are not pre-corrected, which leads to different reliabilities for different individuals and to non-zero “co-reliabilities”. In such cases, the mean of these reliabilities can be used to obtain a single measure of reliability to predict response to selection. Alternatively, the individual specific reliability could be used along with the individual specific selection intensity and generation interval as is done when response to selection is predicted for breeding programs with different “paths” of selection [17, 30].

The additive genetic covariance matrix *Var*(**a**) = **V**_a_ = **A***σ*_*A*_^2^ in (A1) holds covariances between BV of selection candidates, where **A** is the relationship matrix between the selection candidates and *σ*_*A*_^2^ is the additive genetic variance for the selection candidates. If the selection candidates either represent the whole unselected population or are a random sample from such a population, then *σ*_*A*_^2^ is equal to the base additive genetic variance *σ*_*A*,0_^2^ in such a population. The other two components in (A1), *Cov*(**a**, **â**^T^) and *Var*(**â**), depend on *σ*_*A*_^2^ as well as on the properties of the estimator of BV and will be worked out in the following.

Using the standard linear mixed model (1) and assuming known fixed effects (*E*(**y**) = **Xb**) and variance components (*σ*_*A*,0_^2^ *and σ*_*E*_^2^), the EBV can be obtained by regressing BV on the observed phenotypes pre-corrected for fixed effects. The conditional expectation and variance of this regression can be expressed in two equivalent ways [1, 2, 13]:

A2 $$ \begin{array}{rr}\hfill \widehat{\mathbf{a}}=E\left(\mathbf{a}\Big|\mathbf{y}\right)& \hfill =E\left(\mathbf{a}\right)+Cov\left(\mathbf{a},{\mathbf{y}}^{\mathrm{T}}\right)Var{\left(\mathbf{y}\right)}^{-1}\left(\mathbf{y}-\mathbf{X}\mathbf{b}\right),\\ {}\hfill & \hfill ={\mathbf{V}}_{\mathrm{a}}{\mathbf{Z}}^{\mathrm{T}}{\mathbf{V}}_{\mathrm{y}}^{-1}\left(\mathbf{y}-\mathbf{X}\mathbf{b}\right),\\ {}\hfill & \hfill ={\left({\mathbf{V}}_{\mathrm{a}}^{-1}+{\mathbf{Z}}^{\mathrm{T}}{\mathbf{V}}_{\mathrm{e}}^{-1}\mathbf{Z}\right)}^{-1}\mathbf{Z}{\mathbf{V}}_{\mathrm{e}}^{-1}\left(\mathbf{y}-\mathbf{X}\mathbf{b}\right),\end{array} $$

A3 $$ \begin{array}{rr}\hfill \mathrm{V}\mathrm{a}\mathrm{r}\left(\mathbf{a}\Big|\mathbf{y}\right)& \hfill =\mathrm{V}\mathrm{a}\mathrm{r}\left(\mathbf{a}\right)-\mathrm{C}\mathrm{o}\mathrm{v}\left(\mathbf{a},{\mathbf{y}}^{\mathrm{T}}\right)\mathrm{V}\mathrm{a}\mathrm{r}{\left(\mathbf{y}\right)}^{-1}\mathrm{C}\mathrm{o}\mathrm{v}\left(\mathbf{y},{\mathbf{a}}^{\mathrm{T}}\right),\\ {}\hfill & \hfill ={\mathbf{V}}_{\mathrm{a}}-{\mathbf{V}}_{\mathrm{a}}{\mathbf{Z}}^{\mathrm{T}}{\mathbf{V}}_{\mathrm{y}}^{-1}\mathbf{Z}{\mathbf{V}}_{\mathrm{a}},\\ {}\hfill & \hfill ={\left({\mathbf{V}}_{\mathrm{a}}^{-1}+{\mathbf{Z}}^{\mathrm{T}}{\mathbf{V}}_{\mathrm{e}}^{-1}\mathbf{Z}\right)}^{-1}.\end{array} $$

where *Var*(**a**) = **V**_a_ = **A***σ*_*A*,0_^2^ is the additive genetic covariance matrix with respect to the base population, *Var*(**e**) = **V**_e_ = **E***σ*_*E*_^2^ is the residual covariance matrix, and *Var*(**y**) = **V**_y_ = **ZV**_a_**Z**^T^ + **V**_e_ is the phenotypic covariance matrix. The two equivalent expressions are shown to point out that the conditional variance of BV given the phenotypes is the variance of prediction errors (PEV) of EBV, *i.e.*, *Var*(**a**|**y**) = *Var*(**a** − **â**), which is commonly obtained by inverting the coefficient matrix (***V***_*a*_^− 1^ + **Z**^*T*^***V***_*e*_^− 1^**Z**).

Using (A2) it can be shown that the components of (A1), *Cov*(**a**, **â**^T^) and *Var*(**â**), are equal to [1, 2, 13]:

A4 $$ \begin{array}{rr}\hfill Cov\left(\mathbf{a},{\widehat{\mathbf{a}}}^T\right)& \hfill ={\mathbf{V}}_{a,\widehat{a}}={\mathbf{V}}_a{\mathbf{Z}}^T{\mathbf{V}}_y^{-1}\mathbf{Z}{\mathbf{V}}_a\end{array}. $$

A5 $$ \begin{array}{rr}\hfill Var\left(\widehat{\mathbf{a}}\right)& \hfill ={\mathbf{V}}_{\widehat{a}}={\mathbf{V}}_a{\mathbf{Z}}^T{\mathbf{V}}_y^{-1}\mathbf{Z}{\mathbf{V}}_a\end{array}. $$

which along with *Var*(**a**) gives all the required components for computing the individual specific reliabilities in (A1). Since *Cov*(**a**, **â**^*T*^) = *Var*(**â**) and *Var*(**a** − **â**) = *Var*(**a**) − *Var*(**â**) the individual reliabilities in (A1) can be equivalently expressed by contrasting the conditional variance of BV to the variance of BV, *i.e.*, additive genetic variance [1, 2, 13]:

A6 $$ \begin{array}{rr}\hfill {R}^2\left({\widehat{a}}_i\right)& \hfill =\frac{Var\left({\widehat{a}}_i\right)}{Var\left({a}_i\right)}=\frac{Var(a)-Var\left({a}_i-{\widehat{a}}_i\right)}{Var\left({a}_i\right)}=1-\frac{Var\left({a}_i-{\widehat{a}}_i\right)}{Var\left({a}_i\right)}\end{array}. $$

Since the conditional variance of BV, *Var*(*a*_*i*_ − *â*_*i*_), is not affected by selection [1], the expressions (A1) and (A6) give the same reliability when they refer to the same group of individuals, *i.e.*, when the same additive genetic variance is used in both expressions. However, the two expressions do not give the same reliability, if they refer to two different additive genetic variances. Commonly, the base additive genetic variance (*σ*_*A*,0_^2^) is used to compute the base PEV reliability (A6), while the reliability of selection is computed as squared correlation between the EBV and BV in a selected group of individuals (A1) that do not necessarily have the same additive genetic variance (*σ*_*A*_^2^) as the base population.

## Additional files

Additional file 1:
**Theoretical basis of the effect of selection on reliability.** Detailed illustration on how selection reduces the reliability of selection and how this effect differs between types of estimated breeding values [31]. (PDF 7873 kb)
