# Supplementary material for: Reliability of pedigree-based and genomic evaluations in selected populations
Source: Genet Sel Evol. 2015 Aug 14;47(1):65. doi: 10.1186/s12711-015-0145-1 (PMC4536753; doi:10.1186/s12711-015-0145-1)
Supplement: Additional file 1: — Theoretical basis of the effect of selection on reliability. Detailed illustration on how selection reduces the reliability of selection and how this effect differs between types of estimated breeding values [31]. (PDF 7873 kb) [file 12711_2015_145_MOESM1_ESM.pdf]

Theoretical basis of the effect of selection on reliability

Gregor Gorjanc[1], Piter Bijma[2], John M. Hickey[1]

[1]The Roslin Institute and Royal (Dick) School of Veterinary Studies, The University of Edinburgh, Easter Bush, Midlothian, Scotland, UK

[2]Wageningen University, Animal Breeding and Genomics Centre, Wageningen, The Netherlands

The theoretical basis of the effect of selection on reliability has been derived previously by [15] and [16]. These developments are summarized here for completeness and to provide a simple example with initial and equilibrium states. Breeding value of an individual i with known pedigree following the standard quantitative genetic model is:

$$a_i = \tfrac{1}{2}a_s + \tfrac{1}{2}a_d + m_i, \qquad \text{(S1)}$$

where a_s and a_d are respectively the breeding values of sire and dam, and m_i is a Mendelian sampling deviation from the parent average (**BV$_\mathbf{P}$**), $a_{i,P} = \tfrac{1}{2}a_s + \tfrac{1}{2}a_d$. In the notation $a_{i,P}$ letter P denotes that breeding value was computed using only pedigree information. As shown in Appendix 1 the reliability of estimated breeding value (**EBV**, $\hat{a}_i$) using any type of information is defined as a squared correlation between the true and estimated breeding value (**BV**, a_i):

$$R^2(\hat{a}_i) = Corr(a_i, \hat{a}_i)^2 = \frac{Cov(a_i, \hat{a}_i)^2}{Var(a_i)Var(\hat{a}_i)}. \qquad \text{(S2)}$$

The effect of selection on reliability of EBV based on pedigree information only, the estimated parent average, (**EBV$_\mathbf{P}$**) $\left(\hat{a}_{i,P} = \tfrac{1}{2}\hat{a}_s + \tfrac{1}{2}\hat{a}_d\right)$ and EBV based on more than pedigree information $(\hat{a}_i)$ can be demonstrated with an example of truncation

selection. For this purpose, it is important to keep distinction between generations, selection candidates (all individuals in a generation) and selected individuals, and which information is used to obtain the EBV when using (S2). It is assumed throughout that the EBV$_P$ is based only on old information from the parental generation that has been used to select the parents, while the EBV is based on both the old information from the parental generation and the new information from the current generation. Following (S1) the variance of BV$_P$ and BV in progeny in the generation t equals:

$$Var\left(a_{i,P}\right)_t = \tfrac{1}{4}Var(a_s)_{t-1} + \tfrac{1}{4}Var(a_d)_{t-1} + C, \tag{S3}$$

$$Var(a_i)_t = \tfrac{1}{4}Var(a_s)_{t-1} + \tfrac{1}{4}Var(a_d)_{t-1} + Var(m_i)_t + C, \tag{S4}$$

and variance of EBV$_P$ and EBV correspondingly equals:

$$Var\left(\hat{a}_{i,P}\right)_t = \tfrac{1}{4}Var(\hat{a}_s)_{t-1} + \tfrac{1}{4}Var(\hat{a}_d)_{t-1} + \hat{C}, \tag{S5}$$

$$Var(\hat{a}_i)_t = \tfrac{1}{4}Var(\hat{a}_s)_t + \tfrac{1}{4}Var(\hat{a}_d)_t + Var(\widehat{m}_i)_t + \hat{C}, \tag{S6}$$

where C and $\hat{C}$ generically stand for the sum of covariance terms between components and their estimates, respectively. Note that the variance of parental EBV changes when both the old and the new information are used to obtain EBV in the generation t, *i.e.*, $Var(\hat{a}_s)_t$ in (S5) is larger than $Var(\hat{a}_s)_{t-1}$ in (S6). These relationships (S3-6) hold with or without selection when reference to the appropriate groups of individuals is made, *i.e.*, a random set of individuals from the generation $t-1$ become parents of the generation t or a selected set of individuals from the generation $t-1$ become parents of the generation t. Without selection and with random mating $Var(a_s)_{t-1} = Var(a_d)_{t-1} = Var(a)_0$, which is base additive genetic variance. On the other hand,

under the infinitesimal model and ignoring inbreeding $Var(m_i)_t = \frac{1}{2}Var(a)_0$ with or without selection [11]. Departures from this assumption due to drift and selection were quantified empirically in the results. Using these relationships and the property of BLUP that $Cov(a, \hat{a}) = Var(\hat{a})$ [1] in (S2) gives the reliability of EBV$_P$ and EBV for progeny of unselected individuals:

$$R^2\left(\hat{a}_{i,P}\right)_t = \frac{Var\left(\hat{a}_{i,P}\right)_t}{Var(a_i)_t} = \frac{\frac{1}{4}Var(\hat{a}_s)_{t-1}+\frac{1}{4}Var(\hat{a}_d)_{t-1}+\hat{C}}{\frac{1}{4}Var(a_s)_0+\frac{1}{4}Var(a_d)_0+\frac{1}{2}Var(a)_0+C}, \quad \text{(S7)}$$

$$R^2(\hat{a}_i)_t = \frac{Var(\hat{a}_i)_t}{Var(a_i)_t} = \frac{\frac{1}{4}Var(\hat{a}_s)_t+\frac{1}{4}Var(\hat{a}_d)_t+Var(\hat{m}_i)_t+\hat{C}}{\frac{1}{4}Var(a_s)_0+\frac{1}{4}Var(a_d)_0+\frac{1}{2}Var(a)_0+C}. \quad \text{(S8)}$$

Recognizing that denominator in (S7) is equal to $Var(a)_0$ and that $R^2(\hat{a}_i) = \frac{Var(\hat{a}_i)}{Var(a_i)}$ gives the commonly used formula to compute the reliability of EBV$_P$ in progeny of unselected individuals:

$$R^2\left(\hat{a}_{i,P}\right)_t = \frac{1}{4}\left(R^2(\hat{a}_s)_0 + R^2(\hat{a}_d)_0\right), \quad \text{(S9)}$$

which assumes that the covariance between the parental EBV is zero and that parents are not inbred. Deviation from these assumptions can be seen in Table 4 of the manuscript. For example, in the random selection scenario the reliability of EBV in females and males in generation 20 was respectively 0.42 and 0.79, which would according to (S9) give the reliability of EBV$_P$ of 0.30 for progeny in generation 21, while accounting for covariance between the parental EBV and inbreeding gave the reliability of EBV$_P$ of 0.36. In the BLUP selection scenario this deviation was even larger due to more inbreeding in parents caused by selection.

Introduction of selection reduces genetic variability passed to the next generation and this has two effects on the reliability of EBV_P and EBV for progeny of the selected individuals. The first effect is due to reduced variance of EBV_P and EBV in progeny, caused by smaller variance of EBV in the selected individuals:

$$Var(\hat{a})_{t-1,sel} = Var(\hat{a})_{t-1}(1-k), \quad \text{(S10)}$$

where k represents reduction due to selection, $0 < k < 1$ [15, 31]. With truncation selection on normally-distributed EBV, $k = i(i-x)$, where i denotes selection intensity and x the standardized truncation point [31]. For common selection intensities, k is around 0.8. The second effect is due to reduced additive genetic variance in progeny, $Var(a)_t$, caused by smaller additive genetic variance that is passed to the next generation by the selected individuals:

$$Var(a)_{t-1,sel} = Var(a)_{t-1}(1-R^2(\hat{a})_{t-1}k), \quad \text{(S11)}$$

where $R^2(\hat{a})_{t-1}$ is the reliability of EBV of selection candidates [11, 15, 31]. The reliability of EBV_P and EBV in progeny of the selected individuals is then:

$$R^2(\hat{a}_{i,P})_t = \frac{\frac{1}{4}Var(\hat{a}_s)_{t-1,sel}+\frac{1}{4}Var(\hat{a}_d)_{t-1,sel}+\hat{C}}{\frac{1}{4}Var(a_s)_{t,sel}+\frac{1}{4}Var(a_d)_{t,sel}+\frac{1}{2}Var(a)_0+C}, \quad \text{(S12)}$$

$$R^2(\hat{a}_i)_t = \frac{\frac{1}{4}Var(\hat{a}_s)_{t,sel}+\frac{1}{4}Var(\hat{a}_d)_{t,sel}+Var(\widehat{m}_i)_t+\hat{C}}{\frac{1}{4}Var(a_s)_{t,sel}+\frac{1}{4}Var(a_d)_{t,sel}+\frac{1}{2}Var(a)_0+C}. \quad \text{(S13)}$$

Another way to compute reliabilities in the selected population is using the property of BLUP that PEV:

$$Var(a-\hat{a}) = Var(a) - Var(\hat{a}) \quad \text{(S14)}$$

is not affected by selection [1] and equating the reliabilities in base generation and generation t with the corresponding additive genetic variances [15], which gives:

$$R^2(\hat{a}_i)_t = 1 - (1 - R^2(\hat{a}_i)_0)\, Var(a)_0/Var(a)_t. \qquad \text{(S15)}$$

For example, consider a base generation of individuals that are used to generate the next (first) generation without selection, while the second generation is generated from the first generation with a selection of 20% of individuals (Table S1). It is assumed that the same amount of information is available for estimating breeding values in all generations. In the base generation $Var(a_i)_0 = 2.0$, $Var(\hat{a}_i)_0 = 1.5$, and the reliability of EBV of selection candidates is $R^2(\hat{a}_i)_0 = 0.75$. Since there is no selection $Var(a_i)_{0,sel} = Var(a_i)_0$ and $Var(\hat{a}_i)_{0,sel} = Var(\hat{a}_i)_0$. Variances and reliabilities in the first generation of progeny are: $Var(a_i)_1 = Var(a_i)_0 = 2.0$, $Var(\hat{a}_{i,P})_1 = 0.75$, $R^2(\hat{a}_{i,P})_1 = 0.375$, and $R^2(\hat{a}_i)_1 = 0.75$. Selecting 20% of individuals from the first generation of progeny (k ≈ 0.78) reduces variance of EBV in the selected candidates to $Var(\hat{a}_i)_{1,sel} = 0.330$, which consequently reduces variance of EBV$_P$ in the second generation of progeny to $Var(\hat{a}_{i,P})_2 = 0.165$, a reduction of 0.585 in comparison to the unselected case in the first generation. Additive genetic variance in the selected candidates is reduced to $Var(a_i)_{1,sel} = 0.830$ and additive genetic variance in the second generation of progeny is then $Var(a_i)_2 = 1.415$. The reliability of EBV$_P$ in the second generation of progeny is therefore equal to $R^2(\hat{a}_{i,P})_2 = 0.117$, a reduction of 0.258 in comparison to the unselected case in the first generation. Finally, the reliability of EBV in the second generation of progeny equals to $R^2(\hat{a}_i)_2 = 0.647$, a reduction of 0.103 in comparison to the unselected case in the first generation. These reliabilities were computed with

respect to parameters of the second generation. However, if these reliabilities would have been computed from PEV and the base additive genetic variance instead of additive genetic variance in the second generation, the reliability of EBV_P would have been $R^2(\hat{a}_{i,P})_{2,base} = 1 - 1.250/2.000 = 0.375$, an overestimate of 0.258, and the reliability of EBV would have been $R^2(\hat{a}_i)_{2,base} = 1 - 0.500/2.000 = 0.750$, an overestimate of 0.103 (Table S1).

The expressions for the reliability of EBV_P (S12) and EBV (S13) are recursive, using variances from the previous generation. Over several generations of continuous selection an equilibrium is attained and additive genetic variance stabilizes at the so called equilibrium additive genetic variance [15, 11]. The equilibrium reliability of EBV_P and EBV in progeny of selected candidates is then [16]:

$$R^2(\hat{a}_{i,P})_\infty = \frac{1}{2}R^2(\hat{a}_i)_0\left(\frac{1-k}{1+k\left(1-R^2(\hat{a}_i)_0\right)}\right), \quad \text{(S16)}$$

$$R^2(\hat{a}_i)_\infty = R^2(\hat{a}_i)_0\left(\frac{1}{1+k\left(1-R^2(\hat{a}_i)_0\right)}\right). \quad \text{(S17)}$$

Table S1 Variances and reliabilities by generation in the example.

		Generation (t)			
Metric	Eq.	0	1	2	∞
p	/	1.000	0.200	0.200	0.200
k	/	0.000	0.780	0.780	0.780
$Var(a_{i,P})_t$	(S4)	/	1.000	0.415	0.343
$Var(a_i)_t$	(S4)	2.000	2.000	1.415	1.343
$Var(a_i)_{t,sel}$	(S11)	2.000	0.830	0.701	0.685
$Var(\hat{a}_{i,P})_t$	(S5)	/	0.750	0.165	0.093
$Var(\hat{a}_i)_t$	(S6)	1.500	1.500	0.915	0.843
$Var(\hat{a}_i)_{t,sel}$	(S10)	1.500	0.330	0.201	0.185
$Var(a_i - \hat{a}_{i,P})_t$	(S15)	/	1.250	1.250	1.250
$Var(a_i - \hat{a}_i)_t$	(S15)	0.500	0.500	0.500	0.500
$R^2(\hat{a}_{i,P})_t$	(S12)	/	0.375	0.117	0.069
$R^2(\hat{a}_i)_t$	(S13)	0.750	0.750	0.647	0.628

$a_{i,P}, \hat{a}_{i,P}$ – true and estimated breeding value based on pedigree information only, $a_i, \hat{a}_i$ – true and estimated breeding value based on more than pedigree information

These expressions (S16-17) give the same values as the expression (S15). The advantage of expressions (S16-17) is that they can be used without knowing the value of additive genetic variance in the generation of interest, which is commonly unknown. Comparison of (S16) and (S17) over a range of selection intensities shows how selection reduces the equilibrium reliability of EBV_P and EBV (Figure S1). As shown in the example this reduction is greater for the equilibrium reliability of EBV_P than of EBV, *i.e.*, a reduction factor for EBV_P is $(1-k)/c$ and for EBV it is $1/c$, where $c = 1 + k(1 - R^2(\hat{a}_i)_0)$. This difference arises because EBV_P is based on the selected parental EBV that have reduced variance and this old information has low predictive ability of the true BV. At the extreme, when only a single pair of parents is selected, reliability of EBV_P is zero as there is no variation in EBV_P, while BV and EBV of progeny vary due to recombination and segregation of the parental genomes. In the example (Table S1) the reliability of EBV_P in equilibrium is reduced to $R^2(\hat{a}_{i,P})_\infty = 0.069$, a reduction of 0.306, while the reliability of EBV is reduced to $R^2(\hat{a}_i)_\infty = 0.628$, a reduction of 0.122.

When selection intensity and reliabilities are different in male and female selection candidates (S16) can be generalized to [16]:

$$R^2(\hat{a}_{i,P})_\infty = \frac{1}{2}\left(\frac{R^2(\hat{a}_S)_\infty(1-k_S)+R^2(\hat{a}_d)_\infty(1-k_d)}{2}\right), \quad \text{(S18)}$$

and (S17) to:

$$R^2(\hat{a}_s)_\infty = R^2(\hat{a}_s)_0\left(\frac{1+\frac{1}{2}k_d\left(1-R^2(\hat{a}_d)_0/R^2(\hat{a}_S)_0\right)}{1+k\left(1-R^2(\hat{a}_i)_0\right)}\right), \quad \text{(S19)}$$

$$R^2(\hat{a}_d)_\infty = R^2(\hat{a}_d)_0 \left(\frac{1+\frac{1}{2}k_s\left(1-R^2(\hat{a}_s)_0/R^2(\hat{a}_d)_0\right)}{1+\overline{k\left(1-R^2(\hat{a}_\iota)_0\right)}} \right), \tag{S20}$$

where k_s and k_d respectively represent reduction due to selection of sires and dams, and:

$$\overline{k(1-R^2(\hat{a}_\iota)_0)} = \frac{k_s(1-R^2(\hat{a}_s)_0)+k_d\left(1-R^2(\hat{a}_d)_0\right)}{2}. \tag{S21}$$

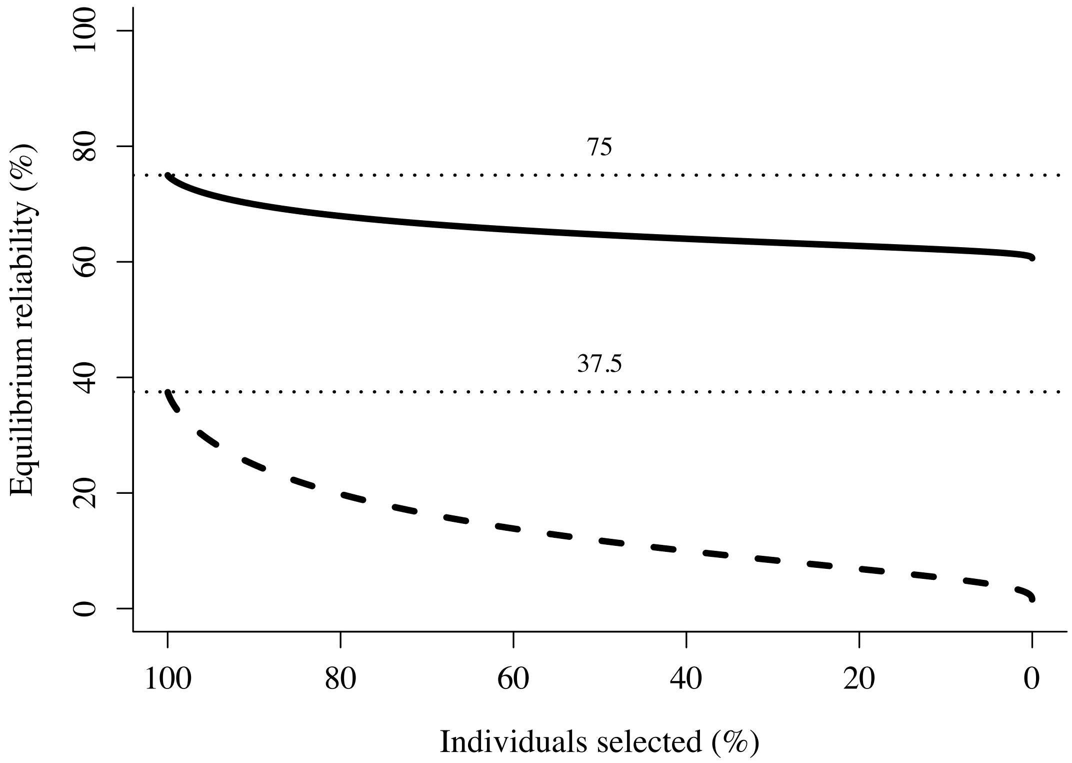

Figure S1 Effect of selection on the equilibrium reliability of the estimated parent average (dashed) and estimated breeding value (solid) in progeny.
